# Supplementary material for: Mixed infections and heteroresistance of Mycobacterium tuberculosis among multidrug-resistant tuberculosis in China: a genomic epidemiology study
Source: Emerg Microbes Infect. 2025 Aug 1;14(1):2534656. doi: 10.1080/22221751.2025.2534656 (PMC12320256; doi:10.1080/22221751.2025.2534656)

**Supplementary Table and figure legends**

**Supplementary** **Table S1 The discrepancies between the phenotypic and genotypic drug resistance by mixed infection, heteroresistance, and treatment outcome**

**Supplementary** **Table S2 Factors associated with poor outcome, based on univariable and multivariable logistic regression modeling.**

**Supplementary Figure S1 The comparison of allele frequency distribution and heterozygous sites between mixed and non-mixed infections.**

The representative plot of allele frequency distribution shows evidence of mixed infections (A-C) and no evidence of mixed infections (D-F). (G) The number of heterozygous sites between mixed and non-mixed infections. ** *P* <0.01. (H) The proportion of heterozygous sites between mixed and non-mixed infections.

**Supplementary Figure S2 The prevalence of genotypic resistance and heteroresistance by drug.**

(A) The bar graph represents the number of isolates with genetic resistance, and the line graph represents the percentage of isolates with heteroresistance-associated variants among patients with resistance-associated variants in different drugs. (B)The allele frequency of all heteroresistance-associated variants by drug, shown with boxplots displaying the minimum, median, interquartile range, and maximum.

**Supplementary Figure S3 The proportion of heteroresistance patients by age.**

Each point represents the proportion of heteroresistance for each age. The red line represents the predicted probability of heteroresistance detection based on a logistic regression model, with age as the predictor variable. The shaded area around the red line represents the 95% confidence interval for the model predictions.

Supplementary Table S1 The discrepancies between the phenotypic and genotypic drug resistance by mixed infection, heteroresistance, and treatment outcome

| Infection type, heteroresistance by WGS and outcome | phenotypically susceptible among genotypically resistant n (%) | | | |  | genotypically susceptible among phenotypically resistant n (%) | | | |
| --- | --- | --- | --- | --- | --- | --- | --- | --- | --- |
|  | RIF | INH | EMB | SM |  | RIF | INH | EMB | SM |
| All outcomes |  |  |  |  |  |  |  |  |  |
| all patients^a^ | 0/920 (0.0) | 0/894 (0.0) | 128/549 (23.3) | 56/652 (8.7) |  | 16/936 (1.7) | 28/922 (3.0) | 31/452 (6.9) | 54/650 (8.3) |
| Mixed infection | 0/99 (0.0) | 0/97 (0.0) | 15/61 (24.6) | 4/74 (5.4) |  | 2/101 (2.0) | 2/99 (2.0) | 3/49 (6.1) | 5/75 (6.7) |
| No evidence of mixed infection | 0/821 (0.0) | 0/797 (0.0) | 113/488 (23.2) | 52/578 (9.0) |  | 14/835 (1.7) | 26/823 (3.2) | 28/403 (6.9) | 49/575 (8.5) |
| heteroresistance | 0/152 (0.0) | 0/150 (0.0) | 30/114 (26.3) | 7/99 (7.1) |  | 2/154 (1.3) | 3/153 (2.0) | 3/87 (3.4) | 9/101 (8.9) |
| No evidence of heteroresistance | 0/768 (0.0) | 0/744 (0.0) | 98/435 (22.5) | 49/553 (8.9) |  | 14/782 (1.8) | 25/769 (3.3) | 28/365 (7.7) | 45/549 (8.2) |
| RIF-heteroresistance | 0/42 (0.0) | 0/40 (0.0) | 8/28 (28.6) | 4/26 (15.4) |  | 0/42 (0.0) | 1/41 (2.4) | 1/21 (4.8) | 3/25 (12.0) |
| INH-heteroresistance | 0/21 (0.0) | 0/21 (0.0) | 6/16 (37.5) | 0/11 (0.0) |  | 0/21 (0.0) | 0/21 (0.0) | 0/10 (0.0) | 3/14 (21.4) |
| EMB-heteroresistance | 0/55 (0.0) | 0/54 (0.0) | 16/55 (29.1) | 4/37 (10.8) |  | 1/56 (1.8) | 1/55 (1.8) | 0/39 (0.0) | 4/37 (10.8) |
| SM-heteroresistance | 0/23 (0.0) | 0/22 (0.0) | 5/18 (27.8) | 3/21 (14.3) |  | 0/21 (0.0) | 0/20 (0.0) | 1/14 (7.1) | 0/18 (0.0) |
| Favorable outcomes^b^ |  |  |  |  |  |  |  |  |  |
| all patients | 0/602 (0.0) | 0/588 (0.0) | 93/359 (25.9) | 43/443 (9.7) |  | 10/651 (1.5) | 19/642 (3.0) | 20/286 (7.0) | 39/439 (8.9) |
| Mixed infection | 0/71 (0.0) | 0/69 (0.0) | 12/37 (32.4) | 4/46 (8.7) |  | 2/69 (2.9) | 2/67 (3.0) | 3/28 (10.7) | 5/47 (10.6) |
| No evidence of mixed infection | 0/531 (0.0) | 0/519 (0.0) | 81/322 (25.2) | 39/397 (9.8) |  | 8/582 (1.4) | 17/575 (3.0) | 17/258 (6.6) | 34/392 (8.7) |
| heteroresistance | 0/94 (0.0) | 0/91 (0.0) | 19/78 (24.4) | 5/60 (8.3) |  | 0/101 (2.9) | 2/100 (3.0) | 1/54 (10.7) | 8/63 (10.6) |
| No evidence of heteroresistance | 0/508 (0.0) | 0/497 (0.0) | 74/287 (25.8) | 38/383 (9.9) |  | 10/550 (1.4) | 17/542 (3.0) | 19/232 (6.6) | 31/376 (8.7) |
| RIF-heteroresistance | 0/36 (0.0) | 0/35 (0.0) | 1/26 (3.8) | 4/22 (18.2) |  | 0/19 (0.0) | 1/19 (5.3) | 0/17 (0.0) | 3/21 (14.3) |
| INH-heteroresistance | 0/16 (0.0) | 0/16 (0.0) | 4/12 (33.3) | 0/9 (0.0) |  | 0/16 (0.0) | 0/16 (0.0) | 0/8 (0.0) | 2/11 (18.2) |
| EMB-heteroresistance | 0/36 (0.0) | 0/34 (0.0) | 11/35 (31.4) | 3/23 (13.0) |  | 0/36 (0.0) | 1/35 (2.8) | 0/24 (0.0) | 3/23 (13.0) |
| SM-heteroresistance | 0/19 (0.0) | 0/18 (0.0) | 5/15 (33.3) | 3/7 (42.9) |  | 0/19 (0.0) | 0/18 (0.0) | 0/10 (0.0) | 0/14 (0.0) |
| Unfavorable outcomes^b^ |  |  |  |  |  |  |  |  |  |
| all patients | 0/112 (0.0) | 0/107 (0.0) | 15/85 (17.6) | 5/86 (5.8) |  | 2/115 (1.7) | 5/113 (4.4) | 2/72 (2.8) | 6/87 (6.9) |
| Mixed infection | 0/11 (0.0) | 0/11 (0.0) | 2/10 (20.0) | 0/9 (0.0) |  | 0/11 (0.0) | 0/11 (0.0) | 0/8 (0.0) | 0/9 (0.0) |
| No evidence of mixed infection | 0/101 (0.0) | 0/96 (0.0) | 13/75 (17.3) | 5/77 (6.5) |  | 2/104 (1.9) | 5/102 (4.9) | 2/64 (3.1) | 6/78 (7.7) |
| heteroresistance | 0/28 (0.0) | 0/28 (0.0) | 2/10 (20.0) | 0/9 (0.0) |  | 1/29 (0.0) | 1/29 (0.0) | 1/18 (5.6) | 0/19 (0.0) |
| No evidence of heteroresistance | 0/84 (0.0) | 0/79 (0.0) | 13/75 (17.3) | 5/77 (6.5) |  | 1/86 (1.9) | 4/84 (4.9) | 1/54 (3.1) | 6/68 (7.7) |
| RIF-heteroresistance | 0/3 (0.0) | 0/2 (0.0) | 0/1 (0.0) | 0/1 (0.0) |  | 0/3 (0.0) | 1/3 (33.3) | 0/1 (0.0) | 0/1 (0.0) |
| INH-heteroresistance | 0/2 (0.0) | 0/2 (0.0) | 1/2 (50.0) | 0/1 (0.0) |  | 0/2 (0.0) | 0/2 (0.0) | 0/1 (0.0) | 0/1 (0.0) |
| EMB-heteroresistance | 0/12 (0.0) | 0/13 (0.0) | 3/13 (23.1) | 0/8 (0.0) |  | 1/13 (1.8) | 0/13 (1.8) | 0/3 (0.0) | 0/8 (10.8) |
| SM-heteroresistance | 0/1 (0.0) | 0/1 (0.0) | 0/1 (0.0) | 0/1 (0.0) |  | 0/1 (0.0) | 0/1 (0.0) | 0/1 (0.0) | 0/1 (0.0) |

^a^ The DST results of EMB for 48 patients and SM for 49 patients were missing.

^b^ The missing outcome data for 170 patients were either lost to follow-up or unable to be evaluated.

Supplementary Table S2. Factors associated with poor outcome^a^, based on univariable and multivariable logistic regression modeling.

| Characteristics | Favorable outcome  (n=651) | Poor outcome  (n=115) | Univariable analysis | | Multivariable analysis | |
| --- | --- | --- | --- | --- | --- | --- |
|  |  |  | OR (95%CI) | *P* | aOR (95%CI) | *P* |
| Sex | | | | | | |
| Female | 190 (29.2) | 16 (13.9) | Reference |  | Reference |  |
| Male | 461 (70.8) | 99 (86.1) | 2.55 (1.47,4.44) | < 0.01 | 1.88 (1.00,3.55) | 0.05 |
| Age | | | | | | |
| 15-29 | 220 (33.8) | 11 (9.6) | Reference |  | Reference |  |
| 30-44 | 182 (28.0) | 20 (17.4) | 2.20 (1.03,4.71) | 0.04 | 2.32 (1.02,5.29) | 0.05 |
| 45-59 | 145 (22.3) | 45 (39.1) | 6.21 (3.11,12.40) | < 0.01 | 4.18 (1.95,8.96) | < 0.01 |
| ≥ 60 | 104 (16.0) | 39 (33.9) | 7.50 (3.69,15.23) | < 0.01 | 5.44 (2.45,12.06) | < 0.01 |
| Migrant | | | | | | |
| No | 329 (50.5) | 80 (69.6) | Reference |  |  |  |
| Yes | 322 (49.5) | 35 (30.4) | 0.45 (0.29,0.68) | < 0.01 |  |  |
| Case detection | | | | | | |
| Self-referral due to symptom | 284 (43.6) | 62 (53.9) | Reference |  | Reference |  |
| Health examination | 31 (4.8) | 0 (0.0) | 0 (0,0) | 1.00 | 0(0,0) | 1.00 |
| Referral | 289 (44.4) | 40 (34.8) | 0.63 (0.41,0.98) | 0.04 | 0.77 (0.46,1.30) | 0.33 |
| Others | 47 (7.2) | 13 (11.3) | 1.27 (0.65,2.48) | 0.49 | 1.65 (0.74,3.70) | 0.22 |
| Sputum smear ^b^ | | | | | | |
| Negative | 189 (29.1) | 19 (16.8) | Reference |  | Reference |  |
| Positive | 460 (70.9) | 94 (83.2) | 2.03 (1.20,3.42) | < 0.01 | 1.51 (0.83,2.75) | 0.18 |
| Previous history of treatment | | | | | | |
| No | 467 (71.7) | 58 (50.4) | Reference |  | Reference |  |
| Yes | 184 (28.3) | 57 (49.6) | 2.49 (1.67,3.73) | < 0.01 | 1.86 (1.15,3.00) | 0.01 |
| Cavity ^c^ | | | | | | |
| No | 327 (55.9) | 50 (46.3) | Reference |  |  |  |
| Yes | 258 (44.1) | 58 (53.7) | 1.47 (0.97,2.22) | 0.07 |  |  |
| Diabetes ^d^ |  |  |  |  |  |  |
| No | 513 (93.8) | 97 (89.0) | Reference |  |  |  |
| Yes | 34 (6.2) | 12 (11.0) | 1.87 (0.93,3.73) | 0.08 |  |  |
| Profiles of drug resistance^e^ | | | | | | |
| MDR/RR | 445 (68.4) | 50 (43.5) | Reference |  | Reference |  |
| Pre-XDR | 170 (26.1) | 51 (44.3) | 2.67 (1.74,4.10) | < 0.01 | 2.18 (1.32,3.60) | < 0.01 |
| XDR | 36 (5.5) | 14 (12.2) | 3.46 (1.75,6.85) | < 0.01 | 3.12 (1.40,6.92) | < 0.01 |
| Lineage 2 | | | | | | |
| No | 67 (10.3) | 5 (4.3) | Reference |  | Reference |  |
| Yes | 584 (89.7) | 110 (95.7) | 2.52 (1.00,6.41) | 0.05 | 1.85 (0.69,5.00) | 0.22 |
| Mixed infection | | | | | | |
| No | 582 (89.4) | 104 (90.4) | Reference |  |  |  |
| Yes | 69 (10.6) | 11 (9.6) | 0.89 (0.46,1.74) | 0.74 |  |  |
| Heteroresistance | | | | | | |
| No | 550 (84.5) | 86 (74.8) | Reference |  |  |  |
| Yes | 101 (15.5) | 29 (25.2) | 1.84 (1.15,2.94) | 0.01 |  |  |

^a^The treatment outcome data for 170 patients is missing.

^b^The sputum smear data for four patients is missing.

^c^The X-ray data for 73 patients is missing.

^d^The diabetes for 110 patients is missing.

^e^MDR/RR-TB patients do not include pre-XDR/XDR-TB patients. Pre-XDR-TB patients do not include XDR patients.

**Supplementary Figure S1 The comparison of allele frequency distribution and heterozygous sites between mixed and non-mixed infections.**

The representative plot of allele frequency distribution shows evidence of mixed infections (A-C) and no evidence of mixed infections (D-F). (G) The number of heterozygous sites between mixed and non-mixed infections. ** *P* <0.01. (H) The proportion of heterozygous sites between mixed and non-mixed infections.


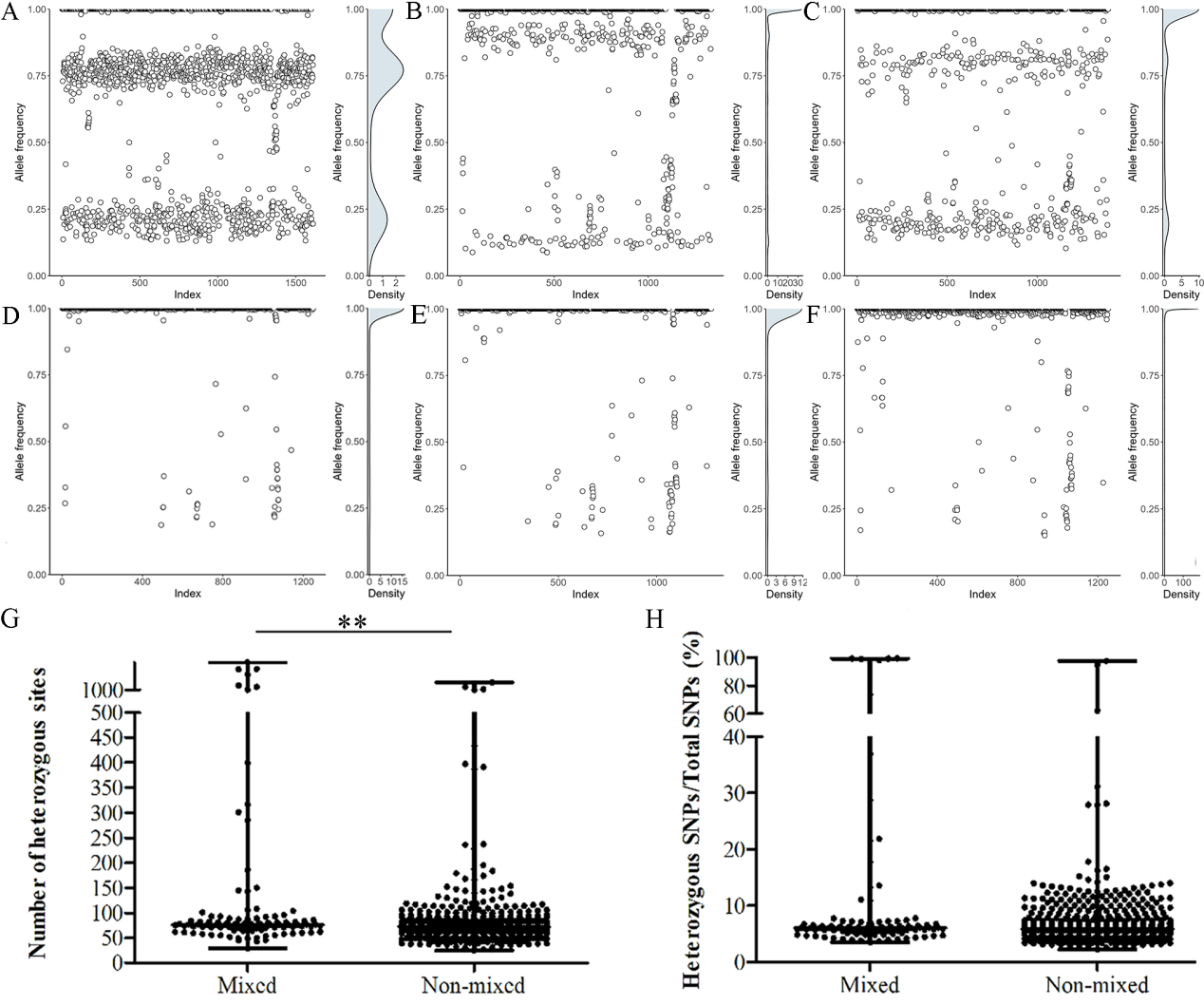


**Supplementary Figure S2 The prevalence of genotypic resistance and heteroresistance by drug.**

(A) The bar graph represents the number of isolates with genetic resistance, and the line graph represents the percentage of isolates with heteroresistance-associated variants among patients with resistance-associated variants in different drugs. (B)The allele frequency of all heteroresistance-associated variants by drug, shown with boxplots displaying the minimum, median, interquartile range, and maximum.


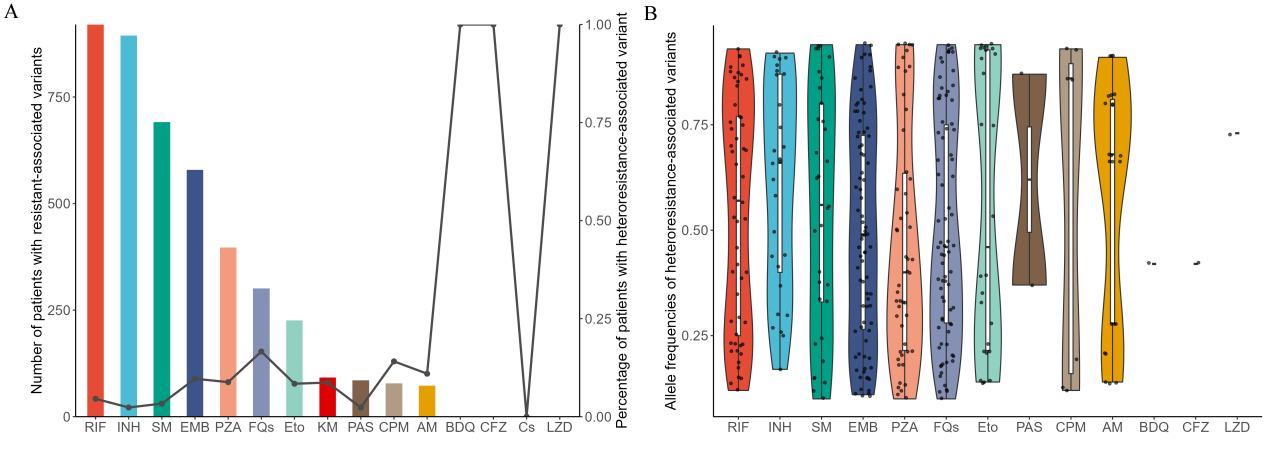


**Supplementary Figure S3 The proportion of heteroresistance patients by age.**

Each point represents the proportion of heteroresistance for each age. The red line represents the predicted probability of heteroresistance detection based on a logistic regression model, with age as the predictor variable. The shaded area around the red line represents the 95% confidence interval for the model predictions.


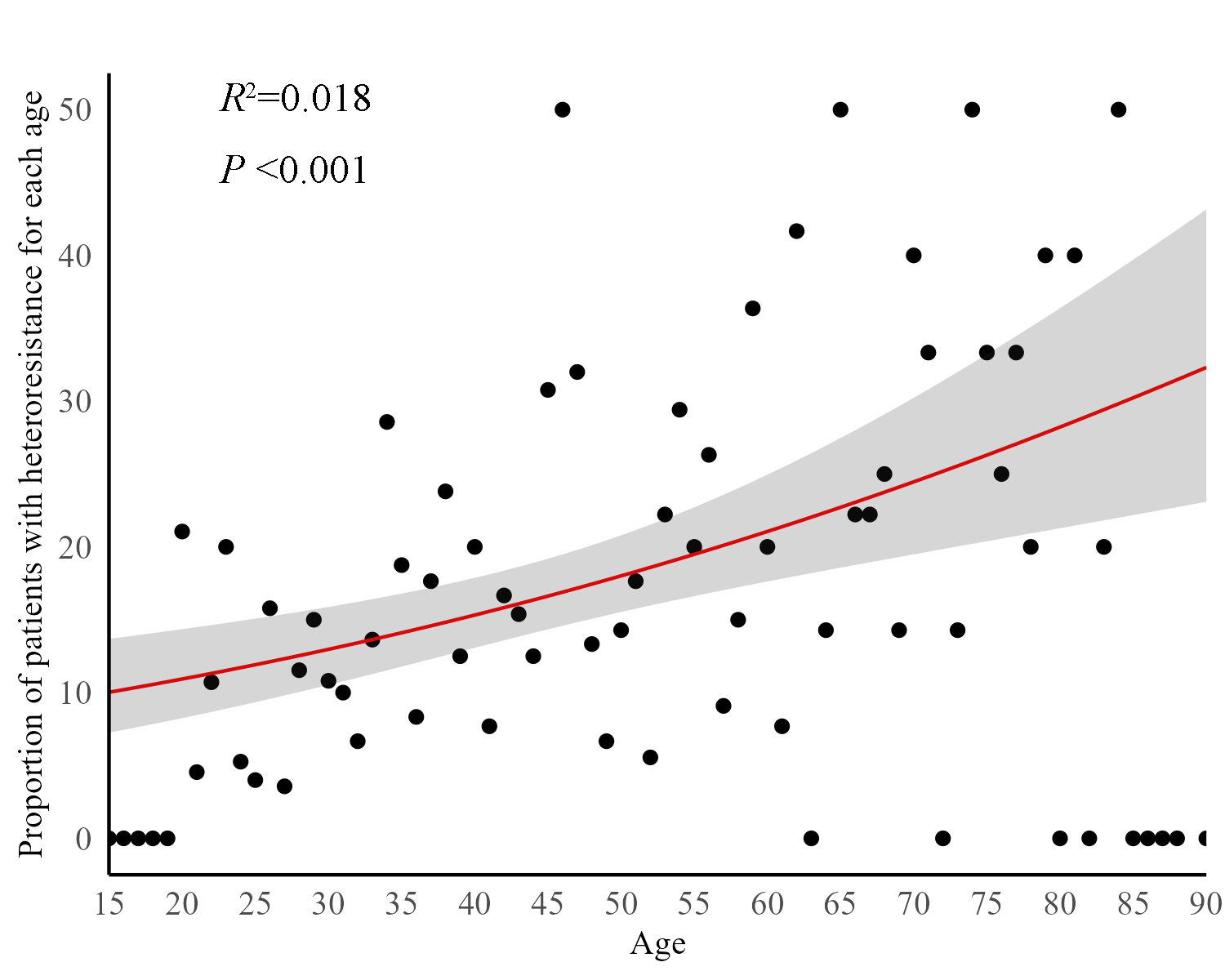

Supplement: Supplementary final.docx [file TEMI_A_2534656_SM3748.docx]
