# Supplementary material for: Mixed infections and heteroresistance of Mycobacterium tuberculosis among multidrug-resistant tuberculosis in China: a genomic epidemiology study
Source: Emerg Microbes Infect. 2025 Aug 1;14(1):2534656. doi: 10.1080/22221751.2025.2534656 (PMC12320256; doi:10.1080/22221751.2025.2534656)
Supplement: manuscript EMI 0529final2 clean.docx [file TEMI_A_2534656_SM3747.docx]

**Mixed infections and heteroresistance of *Mycobacterium tuberculosis* among multidrug-resistant tuberculosis in China: a genomic epidemiology study**

Yanping Liu^1#^, Yangyi Zhang^2#^, Xiaoyu Lu^1^, Zheyuan Wu^2^, Minjuan Li^1^, Rui Zhang^1^, Yixiao Lu^1^, Renjie Hou^1^, Yating Ji^1^, Qingping Zhang^1^, Qi Jiang^3^, Jing Li^2^, Yuan Jiang^2^, Yunxia Wang^4^, Jianhui Yuan^5^, Howard E.Takiff^6^, Xin Chen^7*^, Xin Shen^2*^, Chongguang Yang^1,8*^

^#^Co-authors. The co-authors contributed equally to this article.

*Co-senior authors.

1. School of Public Health (Shenzhen), Shenzhen Key Laboratory of Pathogenic Microbes and Biosafety, Shenzhen Campus of Sun Yat-sen University, Sun Yat-sen University, Guangdong, 518107, China

2. Division of TB and HIV/AIDS Prevention, Shanghai Municipal Center for Disease Control and Prevention, Shanghai, 200336, China

3. Department of Epidemiology and Biostatistics, School of Public Health, Wuhan University, Wuhan, 430071, China

4. Department of Tuberculosis Prevention and Control, Bao’An Chronic Disease Prevention and Cure Hospital in Shenzhen, Shenzhen, 518101, China

5. Nanshan District Center for Disease Control and Prevention, Shenzhen, 518000, China

6. CMBC, Instituto Venezolano de Investigaciones Científicas, IVIC, Caracas, Venezuela

7. Shanghai Municipal Center for Disease Control and Prevention, Shanghai, 200336, China

8. Guangdong Provincial Highly Pathogenic Microorganism Science Data Center, Key Laboratory of Tropical Disease Control, Ministry of Education, Sun Yat-sen University, Guangdong, 518107, China

* Co-senior authors:

Chongguang Yang, School of Public Health (Shenzhen), Shenzhen Campus of Sun Yat-sen University, Shenzhen, 518107, China.

E-mail: yangchg9@mail.sysu.edu.cn

Xin Shen, Division of TB and HIV/AIDS Prevention, Shanghai Municipal Center for Disease Control and Prevention, 1380 Zhongshan West Road, Changning District, Shanghai, 200336, China.

E-mail: [shenxin@scdc.sh.cn](mailto:shenxin@scdc.sh.cn)

Xin Chen, Shanghai Municipal Center for Disease Control and Prevention, 1380 Zhongshan West Road, Changning District, Shanghai, 200336, China.

E-mail: [chenxin@scdc.sh.cn](mailto:chenxin@scdc.sh.cn)**ABSTRACT**

Mixed infection refers to the presence of multiple *Mycobacterium tuberculosis* strains within one host, while heteroresistance denotes the coexistence of drug-susceptible and drug-resistant strains or genotypes. Mixed infections and heteroresistance with *Mycobacterium tuberculosis* can complicate drug resistance diagnosis, treatment options, and transmission inference. We conducted a population-based genomic epidemiological study of multidrug-resistant tuberculosis (MDR-TB) in Shanghai, China, between January 1, 2005, and December 31, 2018, to evaluate the prevalence and impact of mixed infection and heteroresistance on MDR-TB diagnosis and treatment outcomes. Demographic, clinical, and laboratory data were collected, and factors associated with mixed infections and heteroresistance were identified with multivariable logistic regression analysis. Among the 936 MDR-TB patients in our study, 10.8% (101/936) had mixed infections and 16.5% (154/936) exhibited heteroresistance, which was more frequent with second-line anti-TB drugs (*P* < 0.01). There was a higher risk of heteroresistance in older patients (≥ 60 years: aOR 1.91, 95% CI 1.02-3.57), patients with diabetes (2.59, 1.36-4.91), and mixed infections (2.85, 1.67-4.88). Mixed infections and heteroresistance accounted for 22.6% (58/257) of the strains with discrepancies between phenotypic and genotypic drug susceptibility testing (DST). Strains with heteroresistance to EMB had a higher discordance rate than those without (29.1% VS 17.2%, *P* < 0.05). Isolates that were phenotypically susceptible but genotypically resistant harbored minority or low-frequency resistance mutations and were more common in patients with mixed infections and heteroresistance. In summary, mixed infections are significantly associated with heteroresistance, and both mixed infections and heteroresistance can lead to discrepancies between phenotypic and genotypic DST.

**Keywords:** mixed infections; heteroresistance; multidrug-resistant tuberculosis; treatment outcome; whole-genome sequencing

**Introduction**

Tuberculosis (TB), caused by *Mycobacterium tuberculosis* (*M. tuberculosis*), remains one of the most important infectious diseases in terms of global morbidity and mortality. An estimated 10.8 million new TB cases and 1.25 million TB-related deaths occurred worldwide in 2023. Approximately 400,000 people developed multidrug-resistant or rifampicin-resistant TB (MDR/RR-TB), of whom about 29,000 were in China, accounting for 7.3% of the global MDR/RR-TB burden [1]. Although the national TB prevalence has shown a steady decline, the burden of MDR-TB remains a substantial public health concern, complicating efforts to cure TB patients promptly and reduce TB transmission [2,3,4].

More recent studies have identified within-host genetic diversity of *M. tuberculosis*, which may result from mixed infection or within-host microevolution [5-7]. Patients infected with more than one strain of *M. tuberculosis* are said to have mixed infections, which can cause heteroresistance whereby the patient harbors both drug-susceptible (wild genotype) and drug-resistant (drug-resistant mutation) strains [8,9]. Heteroresistance can also arise from a single drug-susceptible *M. tuberculosis* strain if, during within-host evolution, a subpopulation of bacilli acquires drug-resistance mutations [8]. Mixed infections and heteroresistance can cause confusing and discrepant DST profiles, making treatment regimens more complex and potentially less effective [10-13]. The prevalence of mixed infections and heteroresistance is likely underestimated, particularly in patients with MDR-TB [7,14,15].

Although previous studies have explored the impact of mixed infections and heteroresistance on tuberculosis (TB) treatment outcomes [5,9,16-18], findings remain inconsistent regarding their prevalence, influence on resistance detection, clinical implications, and associated risk factors, particularly among patients with MDR-TB. To address these gaps, this study conducted a population-based retrospective analysis of MDR-TB cases in Shanghai, China. By comparing phenotypic drug susceptibility testing (DST) with genotypic resistance predictions derived from whole-genome sequencing (WGS), we aimed to elucidate the relationship between mixed infections, heteroresistance, and treatment outcomes, thereby providing evidence to inform more accurate diagnostics and effective clinical management strategies for MDR-TB.

**Methods**

**Study population**

In recent years, Shanghai has reported an annual incidence rate of approximately 20 cases per 100,000 population, with an estimated 100-150 confirmed MDR-TB cases each year [2]. We conducted a retrospective observational study of culture-confirmed pulmonary multidrug-resistant tuberculosis (MDR-TB) patients diagnosed in Shanghai between January 1, 2005, and December 31, 2018. Patients suspected of having TB are referred to designated local hospitals for diagnosis by sputum smear and culture, and all diagnosed cases are reported to the Shanghai Municipal Centre for Disease Control and Prevention (Shanghai CDC). *M. tuberculosis* strains for this study were collected before the initiation of anti-TB treatment and sent to the Tuberculosis Reference Laboratory at the Shanghai CDC, where DST for first-line drugs rifampicin (RIF), isoniazid (INH), ethambutol (EMB), and streptomycin (SM) was performed using the proportion method [19] on Löwenstein-Jensen medium. Epidemiological information, including demographic data, clinical characteristics, and laboratory results, was obtained from the National Tuberculosis Surveillance System.

**Whole-genome sequencing and drug resistance prediction**

All MDR-TB strains were scraped from Lowenstein-Jensen slants. Their genomic DNA was extracted using the cetyltrimethyl-ammonium bromide-lysozyme (CTAB) method and sequenced on an Illumina Hiseq 2500 platform (Illumina, USA) with a mean depth of 199. The quality of sequence reads was checked using FastQC (v0.11.8) (<https://github.com/s-andrews/FastQC>) and verified paired-end reads were pre-processed with Trimmomatic (v0.39) using the following settings: ILLUMINACLIP=NexteraPE-PE; SLIDINGWINDOW=4 with a trimming threshold=20; Leading=10; Trailing=10; and Minlen=40, as previously described [20]. Paired-end reads were mapped to the reference genome H37Rv (GenBank AL123456.3) using the BWA (v0.7.17) tool. We used SAMtools/BCFtools for SNP-calling with a minimum mapping quality of 30. TB-Profiler (v 5.0.0) was used to predict *M. tuberculosis* lineage and genotypic drug resistance [21,22].

**Detection of mixed infection and heteroresistance**

QuantTB tool was used to detect possible mixed infections [23]. The QuantTB tool identifies and quantifies the abundance of closely related *M. tuberculosis* strains in WGS samples [23]. Heteroresistance was defined by at least ten reads showing resistance-associated variants (RAVs) with an overall frequency between 5% and 95% for all reads at that locus [9,24].

**Definitions**

Migrants were defined as individuals without a Shanghai household registration status. MDR-TB was defined as resistance to at least RIF and INH. Pre-extensively drug-resistant TB (Pre-XDR-TB) was defined as an MDR-TB strain with additional resistance to any fluoroquinolone (FQ) or any of the three second-line injectable drugs: kanamycin (KM), amikacin (AM), and capreomycin (CPM). Extensively drug-resistant tuberculosis (XDR-TB) was defined as an MDR-TB strain with additional resistance to any FQ and any of the three second-line injectable drugs. These definitions were applicable when the study was conducted but were revised in 2021 [25]. Although the definition of XDR changed after 2021, the bedaquiline (BDQ) and linezolid (LZD) were not widely incorporated into standard treatment guidelines for drug-resistant TB in China during the sampling period until 2019, and we adopted the previous definition of MDR-TB to better reflect the drug-resistance patterns relevant to the treatment regimens during the study period. TB patients who were cured or completed treatment were categorized as having a favorable outcome, whereas all other patients were classified as having a poor outcome [18].

**Statistical analysis**

Continuous variables are presented as medians with interquartile ranges (IQRs), while categorical variables are presented as proportions. We used the χ² test and Fisher’s exact test to compare the categorical covariates, and the *t-test* and Wilcoxon test to compare continuous covariates between groups. Variables with *P* < 0.2 in the univariate analyses were assessed by multivariable logistic regression to identify possible risk factors associated with mixed infections and heteroresistance. We used kernel density estimation (KDE) to visualize the density distribution of allele frequencies, employing the “geom_density” function from the ggplot2 package in R. Statistical analyses were performed using R software (version 4.2.0), and a p-value of less than 0.05 was considered statistically significant.

**Ethical approval**

Ethical approval was obtained from the Research Ethics Committee of the School of Public Health (Shenzhen), Sun Yat-sen University (protocol number 2023066). As this was a retrospective study using anonymized data, the requirement for informed consent was waived.

**Results**

**Study population**

We retrospectively collected 1,068 *M. tuberculosis* clinical isolates diagnosed in Shanghai, China, between January 1, 2005, and December 31, 2018, that were identified as MDR/RR by phenotypic DST before treatment initiation. After excluding 19 isolates without genome data and 113 isolates lacking epidemiological information, 936 MDR-TB isolates from an equal number of patients were included for further analysis. These 936 MDR-TB patients had a median age of 40 years (interquartile range, IQR 27-55 years), 72.6% (680/936) were male, and nearly half were migrants (49.3%, 461/936). As described in previous reports from Shanghai [4], most isolates (91.1%, 853/936) belonged to the Beijing family (i.e., Lineage 2). Of the 936 isolates found to be MDR-TB by phenotypic DST, 98.3% (920/936) had genotypic RIF-resistance (RIF-R), 95.5% (894/936) had genotypic INH-resistance (INH-R), and nearly one third (32.2%, 301/936) had genotypic FQs-resistance (FQs-R).

**Identification and characterization of mixed infections**

The QuantTB tool identified 101 (10.8%) MDR-TB patients with infections involving more than one *M. tuberculosis* strain, with the proportion of the majority strain within-host ranging from 51.3% to 89.8%, and the proportion of the minority strain ranging from 10.2% to 48.7%. Most isolates with mixed infections (92.1%, 93/101) belonged to the sublineage 2.2.1. Among these mixed infection patients, the median age was 45 years (IQR, 27-59 years), and males accounted for 78.2% (79/101) (Table 1). In the univariate analysis, the prevalence of comorbidity with diabetes was significantly higher in patients with mixed infections (12.9%, 11/85) than in those infected with a single strain (6.1%, 44/723) (*P <* 0.05) (Table 1). Also, the proportion of patients with XDR-TB was significantly higher in patients with mixed infections compared to those with single-strain infections (12.9% vs 6.0%, *P <* 0.05). In the multivariable logistic regression, however, no statistically significant associations were found with any demographic or clinical characteristics (Table 1).

We then used the density plot to test whether mixed infections contained strains with distinct patterns of allele frequencies. The density plot (Supplementary Figure S1) showed two distinct patterns of SNP read frequencies in mixed infection strains (13/101, 12.9%) that were not prominent in WGS data from non-mixed infection strains (11/835, 1.3%) (*P <* 0.01) (Supplementary Figure S1). Furthermore, the median number of heterozygous sites among all mixed infection strains was also significantly higher than that among infections with only one strain (76 vs. 72, Wilcoxon test, *P <* 0.01) (Supplementary Figure S1).

**The heteroresistance among MDR-TB strains**

Among the 936 MDR-TB isolates, 16.5% (154/936) exhibited heteroresistance with drug-resistant mutations to at least one anti-TB drug. Among these 154 isolates, the median number of drugs showing heteroresistance was two (IQR: 1-5), and heteroresistance was more common with second-line than first-line anti-TB drugs (22.0% vs 10.9%, *P* < 0.01). The proportion of isolates with heteroresistance among those with genotypic resistance was 16.6% (50/301) for FQs, 14.1% (11/78) for CPM resistance, and 8.7% (8/92) for KM (Figure 1A). Two out of 936 isolates were genotypically resistant to BDQ, clofazimine (CFZ), or LZD, and all exhibited heteroresistance (Supplementary Figure S2). Figure 1B shows the distribution of heteroresistant-associated allele frequencies, with a median allele frequency of 50.0% (IQR, 27.0%-80.0%) for heteroresistance mutations. The most commonly observed heteroresistant mutations occurred at sites frequently associated with resistance in clinical isolates, including *embB* M306V (18/56) for EMB, *gyrA* D94G (18/50) for FQs, *rpoB* S450L (13/42) for RIF, and *katG* S315T (9/21) for INH.

We subsequently performed multivariable logistic regression analysis to identify risk factors associated with heteroresistance. The results indicated that older age (45–59 years: adjusted odds ratio [aOR] 2.21, 95% confidence interval [CI]: 1.25–3.90; ≥60 years: aOR 1.91, 95% CI: 1.02–3.57), pre-extensively drug-resistant TB (pre-XDR-TB; aOR 2.39, 95% CI: 1.56–3.67), extensively drug-resistant TB (XDR-TB; aOR 5.20, 95% CI: 2.72–9.94), diabetes (aOR 2.59, 95% CI: 1.36–4.91), and mixed infections (aOR 2.85, 95% CI: 1.67–4.88) were all significantly associated with an increased likelihood of heteroresistance (Table 2 and Supplementary Figure S3).

**The correlation between mixed infections and heteroresistance**

Heteroresistance can result from mixed infections or within-host microevolution of a single strain. In our study, 33.7% (34/101) of mixed infections and 22.1% (34/154) of heteroresistance overlapped (Figure 2A). MDR-TB patients with mixed infections harbored heteroresistance to more drugs than patients infected with a single strain (median (IQR): 5 (1, 5) vs 1 (1, 5), *P* < 0.01) (Figures 2B and C). This difference was prominent for first-line anti-TB drugs (median (IQR): 2 (1, 3) vs 1 (1, 2), *P* < 0.01) but not for second-line drugs (median (IQR): 5 (5, 5) vs 5 (1, 5), *P* = 0.07) (Figure 2D, E).

To further investigate the association of heteroresistance with mixed infections, we examined whether the allele frequencies of heteroresistance-associated alleles correlated with the relative presence of the different strains in mixed infections (Figure 3). Notably, in 12 of the 34 patients (35.3%) with heteroresistance and mixed infections, the allele frequencies of the heteroresistance-associated variants mirrored the distribution of other SNP differences between the two strains present in the sample (Figure 3), showing that the resistance mutations were carried by just one of the strains. In contrast, the allele frequencies of heteroresistance-associated variants in samples containing a single strain were more randomly distributed.

**The impact of mixed infections and heteroresistance on drug-resistance diagnosis and treatment outcomes**

We then compared the resistance profiles from phenotypic DST with the resistance profiles based on the genotypic data and found that 27.5% (257/936) had discrepant results for at least one anti-TB drug. Among these strains, 35.4% (91/257) were phenotypically susceptible and genotypically resistant (P^S^G^R^), 59.5% (153/257) were phenotypically resistant and genotypically susceptible (P^R^G^S^), and 5.1% (13/257) had both types of discrepant results for different drugs. Among the 257 isolates with discrepant DST results, 4.7% (12/257) had mixed infections, 14.0% (36/257) exhibited heteroresistance, and 3.9% (10/257) showed both mixed infections and heteroresistance.

In total, mixed infections or heteroresistance were present in 22.6% (58/257) of the DST discrepancies (Figure 4A, Supplementary Table S1). Most (72.4%, 42/58) of the DST discrepancies in isolates with mixed infections or heteroresistance were P^S^G^R^, and in 90% (38/42) of these, the discrepancy concerned EMB resistance. The discordance rate among strains exhibiting heteroresistance to EMB (29.1%, 16/55) was higher than among strains without heteroresistance to EMB (17.2%, 143/833) (*P* < 0.05). We then examined the frequency distribution of drug-resistant mutations among the P^S^G^R^ patients with genotypic resistance to EMB (Figure 4B) and SM (Figure 4C), identifying minority and low-allele-frequency resistance mutations. Although these included uncommon resistance mutations such as *embB* H312R, *embB* L359I, *embB* A388G, *embB* N399D, and *rrs* n.462C>T, some P^S^G^R^ isolates carried minority populations of *embB* M306V (38.3%, 210 of 549) or *rpsL* K43R (66.6%, 434 of 652), the most common mutations conferring resistance to EMB and SM, respectively.

Finally, we examined factors that might be associated with poor treatment outcomes in patients with MDR-TB (Supplementary Table S2). We found that older patients, males, retreatment patients, and those with pre-XDR/XDR-TB were at a higher risk of poor treatment outcomes (Supplementary Table S2). No significant association was found for either heteroresistance or mixed infections.

**Discussion**

In this population-based study of 936 MDR-TB patients diagnosed in Shanghai, China, between 2005 and 2018, we found that 10.8% had mixed infections and 16.5% exhibited heteroresistance to at least one drug. Furthermore, patients with mixed infections were significantly more likely to harbor heteroresistance. As expected, both mixed infections and heteroresistance were associated with discrepancies between genotypic and phenotypic drug susceptibility testing (DST), which may complicate clinical decision-making and hinder optimal treatment selection.

Patients with mixed infections had a higher risk of harboring heteroresistance because the two strains can have distinct drug resistance profiles [26]. Previous studies have shown that heteroresistance in some patients was associated with mixed infections [4,10], but did not provide genomic evidence correlating the allele frequencies of the heteroresistant-associated mutations with the mixed subpopulations. Our study has provided this evidence by showing that the frequencies of heteroresistant mutations mirror the frequencies of other SNP differences between the two strains. The associations we found between heteroresistance and older patients and patients with diabetes mellitus (DM) suggest that a compromised host immune system may also contribute to the development of heteroresistance. DM can lead to alterations in the immune system, increasing susceptibility to infections [27]. The compromised immune response may impair immune surveillance and bacterial clearance, allowing for immune evasion [27-30]. This facilitates the stable coexistence of polyclonal or heterogeneous *M. tuberculosis* populations, increasing the likelihood of bacterial proliferation and the emergence and persistence of drug-resistance mutations, ultimately manifesting as heteroresistance. This would be consistent with a previous report of increased within-host microevolution of *M. tuberculosis* strains in patients with DM [31,32] and highlights the need for careful management of MDR-TB, particularly among high-risk and immunocompromised populations.

Discrepancies between phenotypic and genotypic DST can result from either novel mutations not included in resistance mutation catalogs (P^R^G^S^), heteroresistance with low frequency minority mutations (P^S^G^R^) or low-level resistance mutations undetected by phenotypic methods (P^S^G^R^) [33,34]. The high rate of discordance between phenotypic and genotypic DST for EMB is likely because the EMB minimum inhibitory concentrations (MICs) for strains carrying the *embB* 306 mutation are very close to the cutoff for defining EMB resistance and can vary slightly in different strains, such that strains carrying *embB* 306 mutations can be phenotypically EMB sensitive [35-37].

Mixed infections and heteroresistance can cause discordance between phenotypic and genotypic drug resistance profiles [12,13,33]. Our study found that discrepant P^S^G^R^ DST results were more common in patients with mixed infections and heteroresistance, who often harbored minority or low-frequency resistance mutations. Undetected minority resistance mutations can compromise the assessment of drug resistance and lead to ineffective TB treatment [38,39]. If only susceptible strains are targeted, resistant strains may survive and proliferate, increasing the risk of treatment failure [12]. Conversely, suppose susceptible strains are overlooked and second-line drugs are used to target the resistant ones. In that case, treatment efficacy may decline due to the weaker bactericidal activity of second-line agents. This can also result in treatment failure, relapse, or the development of further resistance [10]. Therefore, for patients with mixed infections and heteroresistance, combination regimens targeting both susceptible and resistant subpopulations should be considered [10,40]. Meanwhile, such discrepancies could occur during the treatment course and lead to a complex situation [10,12]. Thus, additional frequent monitoring of drug resistance profiles during the treatment course is essential to guide timely and appropriate adjustments to the therapeutic regimen. In summary, when such discrepancies were reported in clinical practice, the potential presence of mixed infection and heteroresistance should be further considered. Although our study did not find a significant association between mixed infections or heteroresistance and poor treatment outcomes, the efficacy of TB treatment can result from the interplay of multiple factors. Additionally, unmeasured potential confounders (e.g., treatment regimen, patient adherence, drug absorption and metabolism, and immune status), as well as the limited sample size, may limit our ability to detect such an association.

This study had several limitations. First, its retrospective design may introduce biases such as selection bias and missing data on comorbidities, sputum smears, X-rays, DSTs, and treatment outcomes, which could affect the interpretation and generalizability of our findings, particularly the association between mixed infections and poor outcomes. Second, MIC testing was unavailable during sample collection, limiting our ability to resolve discordant DST results, especially for borderline resistance. Third, mixed infections were identified using an *in silico* method based on WGS data without additional direct validation, such as isolating distinct strains from cultures, so false positives cannot be ruled out. Lastly, we applied the pre-2021 definitions of pre-XDR/XDR-TB to maintain consistency with clinical practice during the study period. Although this may limit comparability with current standards, analysis using updated definitions consistent results.

In conclusion, this population-based genomic epidemiologic study found that heteroresistance is more common in MDR-TB patients with mixed infections and resistance to second-line drugs. While neither mixed infections nor heteroresistance was associated with unfavorable treatment outcomes in our study, both can lead to discordance between phenotypic and genotypic DSTs. These discrepancies may complicate treatment decisions but also highlight the need for tailored and optimized therapeutic strategies.

**Disclosure statement**

The authors report there are no competing interests to declare.

**Acknowledgment**

This work was supported by the National Key Research and Development Program

of China (2023YFC2307305), the Shenzhen Medical Research Fund (B2303003 and A2302029), the National Natural Science Foundation of China (82373650), Guangdong Natural Science Foundation Project (2024A1515011959), the Shenzhen Nanshan district San-Ming project (SZSM202103008), “Pearl River Talent Plan” Innovation and Entrepreneurship Team Project of Guangdong Province (2021ZT09Y544), the Shenzhen Science and Technology Program (ZDSYS20230626091203007, 2023N042), the Shanghai three-year (2023-2025) action plan to strengthen the public health system (GWVI-11.1-05) and the Top Young Talents in Shanghai.

**Access to data**

The datasets and sequencing data used and analyzed during the current study can be made available upon written request to the corresponding author.

**Contribution**

Y.L., Y.Z., X.S., and C.Y.ang conceived, designed, and managed the study. Y.L., Y.Z., X.L., Z.W., R.Z., M.L., J.L, Y.J., R.H., Y.J., and Q.Z. contributed to the data collection. Y.L., R.Z., and M.L. conducted data cleaning and data analysis. Y.L. drafted the manuscript. Y.L., Y.Z., Y.L., Q.J., Y.W., J.Y., X.C., H.T., X.S. and C.Y. revised the manuscript. All coauthors reviewed and approved the final manuscript before submission.

**Reference**

1. WHO. Global tuberculosis report 2024. Geneva: World Health Organization; 2024.

[2] Chen Y, Shen X, Zhang Y, et al. Impact of financial support on treatment outcomes of multidrug-resistant tuberculosis: A population-based, retrospective cohort study in Shanghai, China. J Clin Tuberc Other Mycobact Dis. 2024;37:100500.

[3] Song HW, Tian JH, Song HP, et al. Tracking multidrug resistant tuberculosis: a 30-year analysis of global, regional, and national trends. Front Public Health. 2024;12:1408316.

[4] Yang C, Luo T, Shen X, et al. Transmission of multidrug-resistant Mycobacterium tuberculosis in Shanghai, China: a retrospective observational study using whole-genome sequencing and epidemiological investigation. Lancet Infect Dis. 2017;17(3):275-284.

[5] Cohen T, Chindelevitch L, Misra R, et al. Within-host heterogeneity of *Mycobacterium tuberculosis* infection is associated with poor early treatment Response: A Prospective Cohort Study. J Infect Dis. 2016;213(11):1796-9.

[6] Nimmo C, Brien K, Millard J, et al. Dynamics of within-host *Mycobacterium tuberculosis* diversity and heteroresistance during treatment. EBioMedicine. 2020;55:102747.

[7] Wyllie D, Do T, Myers R, et al. *M. tuberculosis* microvariation is common and is associated with transmission: Analysis of three years prospective universal sequencing in England. J Infect. 2022.

[8] Hofmann-Thiel S, van Ingen J, Feldmann K, et al. Mechanisms of heteroresistance to isoniazid and rifampin of *Mycobacterium tuberculosis* in Tashkent, Uzbekistan. Eur Respir J. 2009;33(2):368-74.

[9] Shin SS, Modongo C, Baik Y, et al. Mixed *Mycobacterium tuberculosis*-strain infections are associated with poor treatment outcomes among patients with newly diagnosed tuberculosis, independent of pretreatment heteroresistance. J Infect Dis. 2018;218(12):1974-1982.

[10] Abascal E, Herranz M, Ruiz Serrano MJ, et al. In-depth analysis of a mixed *Mycobacterium tuberculosis* infection involving a multidrug-resistant strain and a susceptible strain. Clin Microbiol Infect. 2021;27(4):641-643.

[11] Andersson DI, Nicoloff H, Hjort K. Mechanisms and clinical relevance of bacterial heteroresistance. Nat Rev Microbiol. 2019;17(8):479-496.

[12] van Rie A, Victor TC, Richardson M, et al. Reinfection and mixed infection cause changing *Mycobacterium tuberculosis* drug-resistance patterns. Am J Respir Crit Care Med. 2005;172(5):636-42.

[13] Zetola NM, Shin SS, Tumedi KA, et al. Mixed *Mycobacterium tuberculosis* complex infections and false-negative results for rifampin resistance by GeneXpert MTB/RIF are associated with poor clinical outcomes. J Clin Microbiol. 2014;52(7):2422-9.

[14] Baik Y, Modongo C, Moonan PK, et al. Possible transmission mechanisms of mixed *Mycobacterium tuberculosis* infection in high HIV prevalence country, Botswana. Emerg Infect Dis. 2020;26(5):953-960.

[15] Komakech K, Nakiyingi L, Fred A, et al. Effect of mixed *Mycobacterium tuberculosis* infection on rapid molecular diagnostics among patients starting MDR-TB treatment in Uganda. BMC Infect Dis. 2024;24(1):70.

[16] Chen Y, Jiang Q, Zou J, et al. Deep whole-genome sequencing reveals no evidence for heteroresistance influencing treatment outcomes among drug-susceptible tuberculosis patients. Tuberculosis (Edinb). 2021;130:102120.

[17] Kargarpour Kamakoli M, Sadegh HR, Farmanfarmaei G, et al. Evaluation of the impact of polyclonal infection and heteroresistance on treatment of tuberculosis patients. Sci Rep. 2017;7:41410.

[18] Zetola NM, Modongo C, Moonan PK, et al. Clinical outcomes among persons with pulmonary tuberculosis caused by *Mycobacterium tuberculosis* isolates with phenotypic heterogeneity in results of drug-susceptibility tests. J Infect Dis. 2014;209(11):1754-63.

[19] Xu C, Pang Y, Li R, et al. Clinical outcome of multidrug-resistant tuberculosis patients receiving standardized second-line treatment regimen in China. J Infect. 2018;76(4):348-353.

[20] Gabbassov E, Moreno-Molina M, Comas I, et al. SplitStrains, a tool to identify and separate mixed *Mycobacterium tuberculosis* infections from WGS data. Microb Genom. 2021;7(6):000607.

[21] Coll F, McNerney R, Preston MD, et al. Rapid determination of anti-tuberculosis drug resistance from whole-genome sequences. Genome medicine. 2015;7(1):51.

[22] Phelan JE, O'Sullivan DM, Machado D, et al. Integrating informatics tools and portable sequencing technology for rapid detection of resistance to anti-tuberculous drugs. Genome Med. 2019;11(1):41.

[23] Anyansi C, Keo A, Walker BJ, et al. QuantTB - a method to classify mixed Mycobacterium tuberculosis infections within whole genome sequencing data. BMC Genomics. 2020;21(1):80.

[24] Metcalfe JZ, Streicher E, Theron G, et al. Cryptic microheteroresistance explains *Mycobacterium tuberculosis* phenotypic resistance. Am J Respir Crit Care Med. 2017;196(9):1191-1201.

[25] Sagonda T, Mupfumi L, Manzou R, et al. Prevalence of extensively drug resistant tuberculosis among archived multidrug resistant tuberculosis isolates in Zimbabwe. Tuberc Res Treat. 2014;2014:349141.

[26] Chen Y, Ji L, Liu Q, et al. Lesion heterogeneity and long-term heteroresistance in multidrug-resistant tuberculosis. J Infect Dis. 2021;224(5):889-893.

[27] Ferlita S, Yegiazaryan A, Noori N, et al. Type 2 diabetes mellitus and altered immune system leading to susceptibility to pathogens, especially *Mycobacterium tuberculosis*. J Clin Med. 2019;8(12):2219.

[28] Berezin AE, Kremzer AA, Samura TA, et al. Impaired immune phenotype of circulating endothelial-derived microparticles in patients with metabolic syndrome and diabetes mellitus. J Endocrinol Invest. 2015;38(8):865-74.

[29] Brill KJ, Li Q, Larkin R, et al. Human natural killer cells mediate killing of intracellular *Mycobacterium tuberculosis* H37Rv via granule-independent mechanisms. Infect Immun. 2001;69(3):1755-65.

[30] Kumar NP, Sridhar R, Nair D, et al. Type 2 diabetes mellitus is associated with altered CD8(+) T and natural killer cell function in pulmonary tuberculosis. Immunology. 2015;144(4):677-86.

[31] Allué-Guardia A, García JI, Torrelles JB. Evolution of drug-resistant *Mycobacterium tuberculosis* strains and their adaptation to the human lung environment. Front Microbiol. 2021;12:612675.

[32] Pérez-Navarro LM, Fuentes-Domínguez FJ, Zenteno-Cuevas R. Type 2 diabetes mellitus and its influence in the development of multidrug resistance tuberculosis in patients from southeastern Mexico. J Diabetes Complications. 2015;29(1):77-82.

[33] Chen Y, Takiff HE, Gao Q. Phenotypic instability of *Mycobacterium tuberculosis* strains harbouring clinically prevalent drug-resistant mutations. Lancet Microbe. 2023;4(5):e292.

[34] García-Marín AM, Cancino-Muñoz I, Torres-Puente M, et al. Role of the first WHO mutation catalogue in the diagnosis of antibiotic resistance in *Mycobacterium tuberculosis* in the Valencia Region, Spain: a retrospective genomic analysis. Lancet Microbe. 2024;5(1):e43-e51.

[35] Domínguez J, Boettger EC, Cirillo D, et al. Clinical implications of molecular drug resistance testing for *Mycobacterium tuberculosis*: a TBNET/RESIST-TB consensus statement. Int J Tuberc Lung Dis. 2016;20(1):24-42.

[36] Plinke C, Cox HS, Kalon S, et al. Tuberculosis ethambutol resistance: concordance between phenotypic and genotypic test results. Tuberculosis (Edinb). 2009;89(6):448-52.

[37] Schön T, Juréen P, Giske CG, et al. Evaluation of wild-type MIC distributions as a tool for determination of clinical breakpoints for *Mycobacterium tuberculosis*. J Antimicrob Chemother. 2009;64(4):786-93.

[38] Heyckendorf J, Andres S, Köser CU, et al. What is resistance? Impact of phenotypic versus molecular drug resistance testing on therapy for multi- and extensively drug-resistant tuberculosis. Antimicrob Agents Chemother. 2018;62(2).

[39] Peng Y, Yang C, Li X, et al. Multiple samples improve the sensitivity for detection of mixed Mycobacterium infections. Tuberculosis (Edinb). 2013;93(5):548-50.

[40] Cohen T, van Helden PD, Wilson D, et al. Mixed-strain *mycobacterium tuberculosis* infections and the implications for tuberculosis treatment and control. Clin Microbiol Rev. 2012;25(4):708-19.

**Table and figure legends**

**Table 1 Univariable and Multivariable logistic regression assessing the associated characteristics of mixed infection.**

**Table 2 Factors associated with heteroresistance, based on univariable and multivariable logistic regression modeling.**

**Figure 1 The prevalence of genotypic resistance and heteroresistance by drug.** (A) The bar graph represents the number of isolates with genetic resistance and the line graph represents the percentage of isolates with heteroresistance-associated variants among patients with resistance-associated variants for different drugs. (B)The allele frequency of all heteroresistance-associated variants by drug is shown with boxplots displaying the minimum, maximum, median and interquartile range. Abbreviations: RAVs, resistance-associated variants; RIF, rifampicin; INH, isoniazid; EMB, ethambutol; PZA, pyrazinamide; SM, streptomycin; FQs, fluoroquinolones; Eto, ethionamide; CPM, capreomycin; KM, kanamycin; AM, amikacin.

**Figure 2 The distribution of allele frequency and number of drugs with heteroresistance by mixed infection.** (A) Venn diagram showing overlap of patients with mixed infections and heteroresistance. (B) The number of isolates with heteroresistance stratified by mixed infection or non-mixed infection. (C-E) The density distribution of the number of heteroresistant strains in mixed versus non-mixed infections for: (C) all anti-TB drugs; (D) first-line anti-TB drugs; and (E) second-line anti-TB drugs. Abbreviations: RIF, rifampicin; INH, isoniazid; EMB, ethambutol; PZA, pyrazinamide; SM, streptomycin; FQs, fluoroquinolones; Eto, ethionamide; CPM, capreomycin; KM, kanamycin; AM, amikacin.

**Figure 3 Examples of SNP plots for two clinical samples, illustrating the heteroresistance originated from mixed infections.** The x-axis represents contiguous SNPs across the genome (numbered sequentially), and the y-axis represents the allele frequency for that SNP. The red dots indicate resistant-associated variants. (A) The characteristic pattern of mixed infection isolates with heteroresistance, showing read frequencies and heteroresistant loci clustering into two distinct bands representing the two strains. (B) The pattern of heteroresistant loci in non-mixed infection samples, with read frequencies randomly distributed between 0 and 1.

**Figure 4 The discrepancies between phenotypic and genotypic DST results.** (A) The proportion of patients with discrepancies in DST results across different combinations of mixed infections and heteroresistance, as well as different drugs, stratified by discrepancy types P^R^G^S^ and P^S^G^R^. The P^S^G^R^-associated mutations for ethambutol (B) and streptomycin (C). The pale amber line represents the percentage of strains with the drug resistance-related mutation among strains with genotypic resistance (left Y-axis), while the mocha brown line represents the percentage of strains with the drug resistance-related mutation among strains that are phenotypically sensitive (left Y-axis). Each point represents the allele frequency (right Y-axis) of a specific drug-associated mutation in one patient. Teal blue points represent patients with non-mixed infections, while coral orange points represent patients with mixed infections. A red dashed horizontal line is drawn at y = 0.95, indicating the allele frequency cutoff. Allele frequencies below 0.95 define heteroresistance. Abbreviation: EMB, ethambutol; SM, streptomycin; P^R^G^S^, phenotypically resistant and genotypically susceptible; P^S^G^R^, phenotypically susceptible and genotypically resistant.
